# Quantitative lung ultrasound findings correlate with radial alveolar count in experimental bronchopulmonary dysplasia

**DOI:** 10.1186/s13089-024-00389-y

**Published:** 2024-10-28

**Authors:** Chiara Catozzi, Angelo Modena, Matteo Storti, Francesca Ricci, Gino Villetti, Daniele De Luca

**Affiliations:** 1 Neonatology and Pulmonary Rare Disease Unit, Experimental Pharmacology and Translational Science Department Corporate Preclinical R&D, Chiesi Farmaceutici, Parma, Italy; 2 Division of Pediatrics and Neonatal Critical Care, “A. Beclere” Medical Centre, Paris Saclay University Hospitals, APHP, Paris, France; 3 Physiopathology and Therapeutic Innovation Unit-INSERM U999, Paris Saclay University, Paris, France; 4 Department of Pharmaceutical Sciences, University of Piemonte Orientale, Novara, Italy

**Keywords:** Lung ultrasonography, Neonate, Alveolarization, Prematurity

## Abstract

We investigated the relationship between the degree of alveolarization and ultrasound-assessed lung aeration in a validated preterm rabbit model of experimental bronchopulmonary dysplasia (BPD). Lung ultrasound findings were heterogeneously abnormal and consisted of zones with interstitial, interstitial-alveolar or consolidated patterns. The median radial alveolar count was 10.1 [8.4–11.5], 7.8 [6.1–9] and 7.3 [1.8–10.1] in rabbits with interstitial, interstitial-alveolar or consolidated ultrasound pattern, respectively (overall *p* = 0.036). Alveolar count and lung ultrasound score were significantly correlated (ρ = − 0.044 (95%CI: − 1; − 0.143), *p* = 0.009; τ_-b_ = − 0.362 (95%CI: − 0.6; − 0.1), *p* = 0.017).

## Introduction

Bronchopulmonary dysplasia (BPD) is characterized by altered alveolar and vascular development due to the abnormal reparative responses to pre-/post-natal injuries and is a multifactorial and evolutive disorder [1]. This translates in a chronic respiratory failure representing a *continuum* starting from early life, when BPD is evolving, until late infancy, where its chronic consequences are evident: this is well described by the “chronic pulmonary insufficiency of prematurity” concept [1]. The rabbit model of experimental BPD encompasses several of these features as animals are delivered prematurely and exposed to different level of hyperoxia to induce an alveolarization impairment similar to mechanisms occurring in human infants with evolving BPD [2].

Lung ultrasound can detect abnormalities in infants with evolving BPD yet in its early phase (i.e. at 7–14 days of life [3]) and the associated loss of lung aeration can be followed up to titrate the respiratory support and predict the diagnosis of BPD at 36 weeks post-menstrual age [4]. Lung ultrasound findings are similar in infants with evolving BPD and in rabbits with experimental BPD [5]. The evaluation of lung aeration by quantitative lung ultrasound has been validated against a number of techniques including CT-scan and gas exchange measures in various patients and setting [6]. Nonetheless, we lack data regarding the ultrasound-assessed lung aeration and the impaired alveolarization typical of BPD. We planned to fill this gap using the rabbit model of experimental BPD and we hypothesize that lung aeration would be correlated to the degree of alveolarization.

## Methods

We used the experimental model of BPD based on preterm rabbits delivered at 28 days of gestation (i.e. equivalent to the saccular stage of human lung development [2]) and exposed to hyperoxia shortly after birth [5]. In details, rabbits were exposed to two degrees of hyperoxia with different inspired oxygen fraction (FiO_2_), that is: moderate (FiO_2_ = 0.7, n = 6) or severe hyperoxia (FiO_2_ = 0.95, n = 9). Hyperoxia was provided right after birth for 1 h at 60% relative humidity. Two additional groups of animals treated in normoxia (FiO_2_ = 0.21) were also studied: one consisted of preterm rabbits (n = 8) and another was a control group of term (42 days of gestation) healthy rabbits (n = 5). The rest of animal care and treatment was provided as previously described [5].

**Fig. 1:**
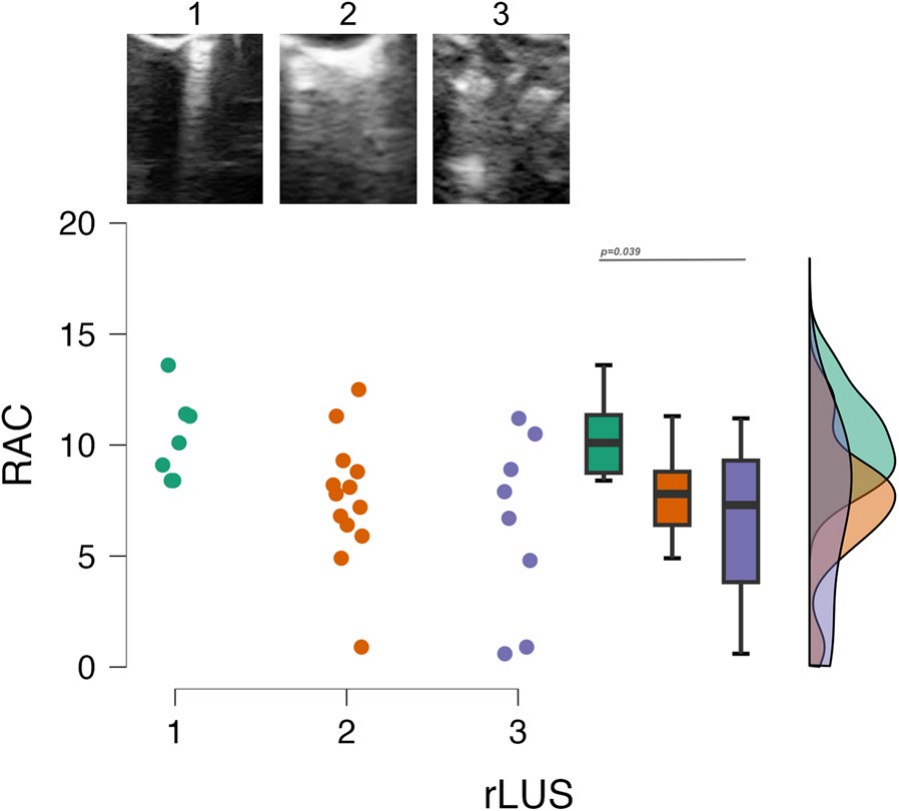
Relationship between radial alveolar count (RAC) and quantitative lung ultrasound findings. Green, orange, and violet dots represent individual RAC data for each rabbit with interstitial (rLUS = 1), interstitial-alveolar (rLUS = 2) or consolidated (rLUS = 3) ultrasound pattern, respectively. Illustrative pictures of lung ultrasound pattern are shown above the dot graph. Box plots depict (from top to bottom) maximum, 75th, 50th, 25th, and minimum RAC values. The curves on the right-side represent the density (distributions) of data points. No rabbit had rLUS = 0. Data were analyzed with Kruskal–Wallis test (overall *p* = 0.036, n = 28). The horizontal line represents the significant post hoc comparisons given by Dunnett test. RAC and rLUS are dimensionless variables. *RAC* radial alveolar count, *rLUS* rabbit lung ultrasound score

Lung ultrasound was performed in newborn rabbits at on the 7th and 14th day of postnatal life, for severe and moderate hyperoxia groups, respectively; pups exposed to severe hyperoxia always die after the first week of life, thus they could not be scanned at the 14th days. Preterm rabbits exposed to normoxia and control healthy rabbits also underwent lung ultrasound on the 14th day of life. Lung ultrasound was performed as previously described [5]: in detail, each hemithorax was scanned as a whole, using a micro-linear high-frequency (20 MHz) probe (Fujifilm Visualsonics inc, Toronto-ON, Canada), and rabbit lung ultrasound score (rLUS) was calculated, by averaging results from the two hemithoraces. rLUS was based on the classical lung ultrasound semiology assigning a 0-to-3 score to the observed ultrasound patterns (Fig. 1) [6]. The evaluation of rLUS is known to have a high inter-rater agreement [5].

After the ultrasound scan, rabbits were euthanatized as previously described [5]. In detail, lungs were removed and fixed with 10% buffered formalin under constant pressure for 24 h, then transferred to 70% ethanol, embedded in paraffin, and stained with eosin and hematoxylin following standard histology procedures. A section for each lung was used to calculate the radial alveolar count (RAC) in 40 fields/section [7]. Similarly to the rLUS calculation, the values of the right and left lung were averaged.

Research protocol was approved by the Italian Ministry of Health review board (n. 875/2021-PR) and met all relevant regulations about animal research. ARRIVE guidelines were followed [8]. A convenience sample size was decided based on previous studies about lung mechanics and pathology on the same experimental model [9]. RAC and rLUS were analyzed with Spearman (ρ) and Kendall (τ_-b_) correlation coefficients. RAC values were expressed as median [25th–75th percentile] and compared between rabbits with different ultrasound pattern using Mann-Withney test followed by Dunnett post hoc test. JASP 0.17.1 (JASP Team 2023) was used and *p*-values <0.05 were considered significant.

## Results

**Fig. 2:**
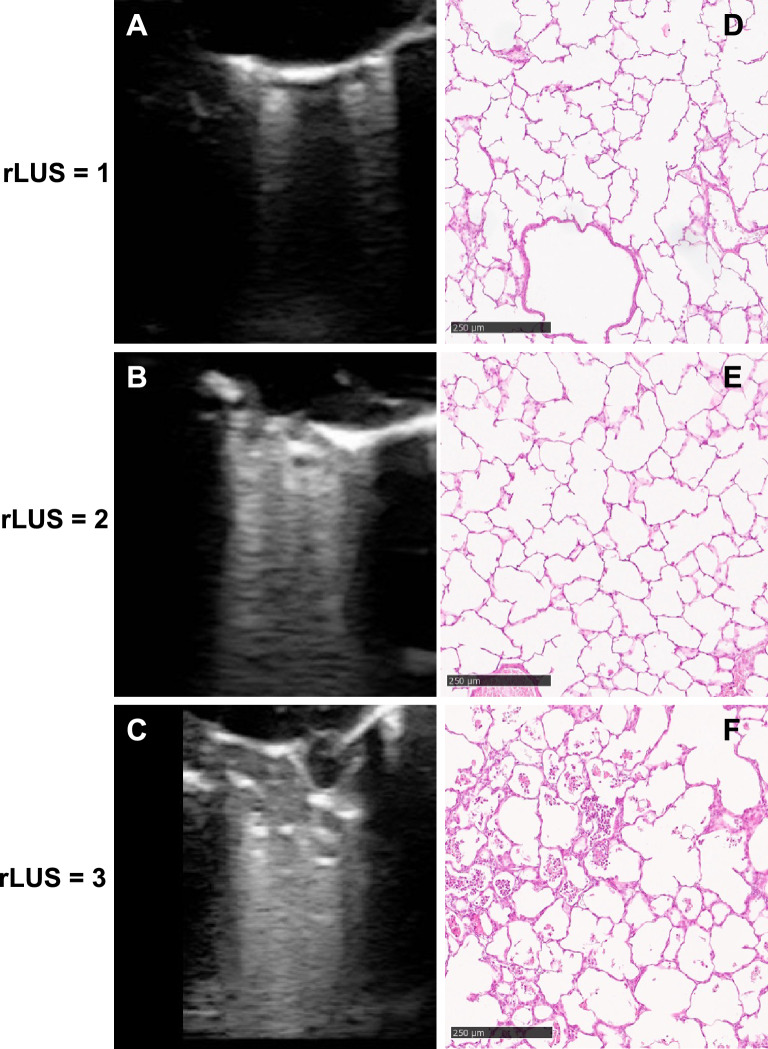
Illustrative pictures of corresponding ultrasound and histology findings. Panels **A**, **B** and **C** show interstitial, interstitial-alveolar or consolidated ultrasound patterns, respectively. Panels **D**, **E** and **F** show the corresponding histology findings image where RAC has been calculated (eosin and hematoxylin, magnification X10). No rabbit had rLUS = 0. RAC and rLUS are dimensionless variables. *RAC* radial alveolar count, *rLUS* rabbit lung ultrasound score

Lung ultrasound findings were heterogeneously abnormal in hyperoxia-exposed preterm rabbits and consisted of zones with interstitial (rLUS = 1) or interstitial-alveolar (rLUS = 2) pattern, and others with consolidations (i.e. total loss of aeration; rLUS = 3). No rabbit had a completely normal lung ultrasound appearance. RAC was 10.1 [8.4–11.5], 7.8 [6.1–9] and 7.3 [1.8–10.1] in rabbit pups with interstitial, interstitial-alveolar or consolidated ultrasound pattern, respectively (overall *p*=0.036, details and post hoc comparisons in Fig.1). RAC and rLUS were significantly correlated with a ρ = − 0.044 (95%CI: − 1; − 0.143; *p*=0.009) and a τ_-b_ = − 0.362 (95%CI: − 0.6; − 0.1; *p*=0.017). Figure 2 shows illustrative images of matched ultrasound and histology findings.

## Discussion

Quantitative lung ultrasound describes lung aeration (i.e. the open lung volume available for gas exchange), which can be acutely affected by several pathological processes such as local inflammation, airway obstruction or pleural effusion typical of acute respiratory failure [6]. BPD is different because its gas exchange impairment is slowly developing, becomes chronic and is characterized by an arrested lung development leading to an abnormal alveolarization [1]. We demonstrated that, in an experimental model of BPD, the ultrasound-assessed lung aeration and the alveolarization process (as estimated by RAC) are correlated. This means that the histologically altered lung development can lead to a relevant loss of lung aeration which may be captured by quantitative lung ultrasound. Thus, the impaired alveolarization typical of BPD, when enough severe, might have a corresponding ultrasound imaging.

This had been suspected with simpler histology measures [5] but was not demonstrated before, as lung ultrasound is a relatively new technique in neonatology, and it is obviously difficult to have access to lung biopsy from patients. The use of a well-standardized experimental model was needed, and our study fills this knowledge gap. In fact, our finding provides the pathobiological support for the use of lung ultrasound to predict the diagnosis of BPD and monitor the evolution of chronic respiratory insufficiency of prematurity as it has been done in patients with evolving or established BPD [4, 10]. Nonetheless, the data do not demonstrate that lung ultrasound has a sufficient resolution to distinguish the effect of potential therapies to be investigated in this animal experimental model.

The work has limitations inherent to the use of an animal model as previously described [5]. However, given the difficult access to human histology specimens, our data represent a significant addition to the current knowledge. The used sample size is also relatively small but is similar to populations enrolled in previous studies [5] and the homogeneous and well-standardized model guarantees the solidity of our data. In conclusion, in an experimental model of BPD, ultrasound-assessed lung aeration and the degree of alveolarization are correlated.

## Data Availability

The datasets used and/or analyzed during the current study are available from the corresponding author on reasonable request, upon an agreement for research purposes, and respecting all the relevant regulations.

## Abbreviations

BPD: Bronchopulmonary dysplasia
FiO2: Inspired oxygen fraction
RAC: Radial alveolar count
rLUS: Rabbit lung ultrasound score

## References

[1] Steinhorn R, Davis JM, Göpel W, Jobe A, Abman S, Laughon M et al (2017) Chronic pulmonary insufficiency of prematurity: developing optimal endpoints for drug development. J Pediatr 191:15-21.e1 29173299 10.1016/j.jpeds.2017.08.006

[2] D’Angio CT, Ryan RM (2014) Animal models of bronchopulmonary dysplasia. The preterm and term rabbit models. Am Physiol Lung Cell Mol Physiol 307:L959–L969 10.1152/ajplung.00228.2014 25326582

[3] Loi B, Vigo G, Baraldi E, Raimondi F, Carnielli VP, Mosca F et al (2021) Lung ultrasound to monitor extremely preterm infants and predict BPD: multicenter longitudinal cohort study. Am J Respir Crit Care Med 203:1398–1409. 10.1164/rccm.202008-3131OC 33352083 10.1164/rccm.202008-3131OC

[4] Pezza L, Alonso-Ojembarrena A, Elsayed Y, Yousef N, Vedovelli L, Raimondi F et al (2022) Meta-analysis of lung ultrasound scores for early prediction of bronchopulmonary dysplasia. Ann Am Thorac Soc 19:659–667. 10.1513/AnnalsATS.202107-822OC 34788582 10.1513/AnnalsATS.202107-822OC

[5] Loi B, Casiraghi C, Catozzi C, Storti M, Lucattelli M, Bartalesi B et al (2021) Lung ultrasound features and relationships with respiratory mechanics of evolving BPD in preterm rabbits and human neonates. J Appl Physiol 131:895–904 34292788 10.1152/japplphysiol.00300.2021

[6] Mongodi S, De Luca D, Colombo A, Stella A, Santangelo E, Corradi F et al (2021) Quantitative lung ultrasound: technical aspects and clinical applications. Anesthesiology 134:949–965. 10.1097/ALN.0000000000003757 33819339 10.1097/ALN.0000000000003757

[7] Cooney TP, Thurlbeck WM (1982) The radial alveolar count method of Emery and Mithal: a reappraisal 2–intrauterine and early postnatal lung growth. Thorax 37:580–583 7179186 10.1136/thx.37.8.580 PMC459378

[8] Percie du Sert N, Hurst V, Ahluwalia A, Alam S, Baker MT et al (2020) The ARRIVE guidelines 2.0: updated guidelines for reporting animal research. PLOS Biol 18(7):e3000410. 10.1371/journal.pbio.3000410 32663219 10.1371/journal.pbio.3000410 PMC7360023

[9] Richter J, Toelen J, Vanoirbeek J, Kakigano A, DeKoninck P, Verbeken E et al (2014) Functional assessment of hyperoxia-induced lung injury after preterm birth in the rabbit. Am J Physiol Lung Cell Mol Physiol 306:L277–L283 24375793 10.1152/ajplung.00315.2013

[10] Savoia M, Miletic P, De Martino M, Morassutti FR (2022) Lung ultrasound score follows the chronic pulmonary insufficiency of prematurity trajectory in early infancy. Eur J Pediatr 181:4157–4166 36166097 10.1007/s00431-022-04629-y

